# Trapped Ion Mobility Spectrometry and Parallel Accumulation–Serial Fragmentation in Proteomics

**DOI:** 10.1016/j.mcpro.2021.100138

**Published:** 2021-08-17

**Authors:** Florian Meier, Melvin A. Park, Matthias Mann

**Affiliations:** 1 Department Proteomics and Signal Transduction, Max Planck Institute of Biochemistry, Martinsried, Germany; 2 Functional Proteomics, Jena University Hospital, Jena, Germany; 3 Bruker Daltonics Inc, Billerica, Massachusetts, USA

**Keywords:** mass spectrometry, TOF, ion mobility, TIMS, PASEF, data acquisition, collisional cross section, CCS, collisional cross section, DC, direct current, dda, data-dependent acquisition, dia, data-independent acquisition, IMS, ion mobility spectrometry, MS/MS, tandem MS, PASEF, parallel accumulation–serial fragmentation, QTOF, quadrupole TOF, TIMS, trapped ion mobility spectrometry

## Abstract

Recent advances in efficiency and ease of implementation have rekindled interest in ion mobility spectrometry, a technique that separates gas phase ions by their size and shape and that can be hybridized with conventional LC and MS. Here, we review the recent development of trapped ion mobility spectrometry (TIMS) coupled to TOF mass analysis. In particular, the parallel accumulation–serial fragmentation (PASEF) operation mode offers unique advantages in terms of sequencing speed and sensitivity. Its defining feature is that it synchronizes the release of ions from the TIMS device with the downstream selection of precursors for fragmentation in a TIMS quadrupole TOF configuration. As ions are compressed into narrow ion mobility peaks, the number of peptide fragment ion spectra obtained in data-dependent or targeted analyses can be increased by an order of magnitude without compromising sensitivity. Taking advantage of the correlation between ion mobility and mass, the PASEF principle also multiplies the efficiency of data-independent acquisition. This makes the technology well suited for rapid proteome profiling, an increasingly important attribute in clinical proteomics, as well as for ultrasensitive measurements down to single cells. The speed and accuracy of TIMS and PASEF also enable precise measurements of collisional cross section values at the scale of more than a million data points and the development of neural networks capable of predicting them based only on peptide sequences. Peptide collisional cross section values can differ for isobaric sequences or positional isomers of post-translational modifications. This additional information may be leveraged in real time to direct data acquisition or in postprocessing to increase confidence in peptide identifications. These developments make TIMS quadrupole TOF PASEF a powerful and expandable platform for proteomics and beyond.

## Highlights

• TIMS is a compact, highly efficient, and flexible ion mobility device.
• PASEF increases sequencing speed without compromising sensitivity.
• Collisional cross-section information remains to be fully explored and utilized.

## Graphical Abstract

**Figure:**
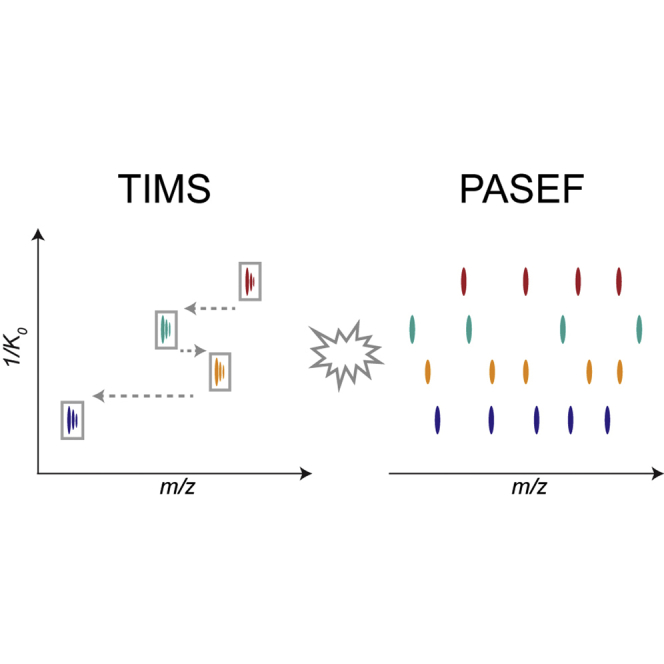

## In Brief

The combination of ion mobility and MS is increasingly attractive in the field of proteomics research. Here, we provide a perspective on the recent development of TIMS and PASEF. TIMS offers an additional dimension of separation and collisional cross-section information, whereas PASEF greatly increases the efficiency of MS/MS experiments. As the user base broadens and computational tools evolve, a wide range of applications emerge from structural to single-cell proteomics.

As of today, the Human Proteome Project has accumulated MS-based evidence for the expression of >90% of the ∼20,000 predicted protein-coding human genes (1), and hundreds of thousands of proteins have been mapped across the kingdoms of life (2). Nevertheless, in particular for complex mammalian proteomes, the analysis depth in single experiments remains limited by chromatographic peak capacity as well as speed, sensitivity, and dynamic range of the mass analyzer (3, 4, 5, 6, 7, 8). State-of-the-art proteomics workflows measure the chromatographic retention time and mass of peptide and fragment ions (9). Integrating ion mobility spectrometry (IMS) adds an extra dimension that separates ions by their size and shape in the gas phase (10, 11). In classic drift tube IMS, ions migrate through an inert buffer gas under the influence of a weak electric field, whereas collisions with buffer gas molecules retard the progress of the ions. As larger ions have more collisions with the gas, they are more strongly retarded than their smaller counterparts. Thus, smaller ions, having a smaller cross section, arrive earlier at the detector than ions with a larger collisional cross section (CCS). The ion mobility *K* is then defined as the ratio of the analyte's steady-state net drift velocity to the applied electric field, and it is convention to calculate the reduced ion mobility *K*_*0*_ at standard pressure and standard temperature, often reported as the inverse reduced ion mobility *1/K*_*0*_ (12).

Coupling IMS to MS detectors dates back to the 1960s, whereas much of the groundwork of today's IMS devices was laid in the 1980s and 1990s (reviewed in Refs. (13, 14)). Since then, a variety of IMS methods have been developed and gradually became available beyond specialized laboratories (15). This comprises methods separating ions in time (similar to drift tubes), for example, traveling wave IMS (16, 17), as well as spatially dispersive methods that filter ions based on their mobility, such as high-field asymmetric waveform IMS (18, 19). The millisecond timescale of the analysis and its complementary selectivity are key features that make IMS potentially useful in proteomics research (20, 21, 22, 23, 24, 25, 26). However, until recently, the complexity of the instrumentation and data, as well as poor sensitivity, had prevented widespread use. The latest-generation IMS devices have greatly increased ion transmission and ease of use, and a plethora of exciting application areas is now emerging (27, 28, 29, 30, 31). In this perspective, we focus on the trapped ion mobility spectrometry (TIMS) (32, 33) and parallel accumulation–serial fragmentation (PASEF) technology (34), which we believe holds particular potential for dramatically extending the reach of proteomics.

## Development of TIMS and PASEF

**Fig. 1:**
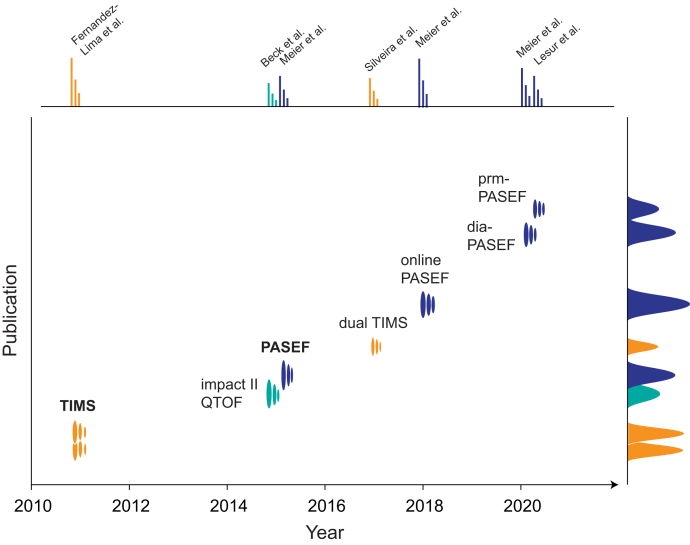
**Timeline of selected milestones in the historic development of TIMS and PASEF acquisition modes depicted as a TIMS data structure with arbitrary peak heights** (29, 32, 33, 34, 36, 37, 38, 39)**.** PASEF, parallel accumulation–serial fragmentation; QTOF, quadrupole TOF; TIMS, trapped ion mobility spectrometry.

In the perspective of the long history of ion mobility, TIMS and PASEF are relatively new techniques that emerged only over the last 10 years (Fig. 1). In 2011, Fernandez-Lima *et al.* (32, 33) introduced TIMS, which reversed the concept of classical drift tube IMS. Rather than moving ions through a stationary gas, TIMS holds ions stationary against a moving gas and then releases them according to their mobility. Most importantly, this renders the ion mobility resolution independent of the physical dimensions of the device and allows a compact design, operating at an order of magnitude lower voltages, and providing more versatile operation modes than prior IMS analyzers (35). The current commercial configuration features a dual TIMS device, in which the first part of an ∼10 cm ion tunnel is operated as an ion storage device in series with TIMS analysis in the second part (29). Because of this capture and release cycle, this configuration can use up to 100% of incoming ions (36).

Since TIMS requires only 100 ms to analyze a wide ion mobility range, producing mobility peaks as narrow as 1 to 2 ms full width at half maximum, it is advantageous to integrate it with fast detection such as TOF mass analyzers. As these can acquire ∼10 spectra per millisecond, TOF analyzers can readily follow the progress of a TIMS analysis—producing several spectra across a single ion mobility peak. With the *impact II*, Bruker had already marketed a quadrupole TOF (QTOF) platform with a mass resolution of >35,000 across the full *m/z* range for proteomics applications (37). The instrument features two electrodynamic funnels and efficient ion optical elements to transfer >80% of the ions entering the vacuum through quadrupole and collision cell. The latter was designed for high MS/MS rates and serves as an ion storage for the time of a single TOF pulse to ensure a high duty cycle for the *m/z* range of interest, even when operated with continuous electrospray ion sources. This platform then became the basis of the next-generation *timsTOF* instruments equipped with a TIMS device in the first vacuum stage.

The basic idea of PASEF is to utilize the accumulation and ordered “serial” release feature of TIMS to increase the efficiency of MS/MS experiments (34). We achieved this by first accumulating ions “in parallel” to their mobility analysis so as to avoid ion losses and then by synchronizing the precursor selection in the analytical quadrupole with the elution of mobility-separated ions from the TIMS device. Rather than selecting only a single precursor per TIMS scan, PASEF selects multiple precursors one after another, that is, serially during each TIMS scan. Because the precursor signal is compressed and serially eluted in narrow and time-separated ion mobility peaks, the selection and fragmentation occur without loss in sensitivity. First demonstrated manually on a prototype instrument, online implementation required further development of the instrument electronics and firmware to handle the data in real time and to switch the quadrupole position rapidly on a submillisecond timescale. To date, and as discussed further later, the PASEF principle has been successfully implemented with data-dependent (29), targeted (38), as well as data-independent acquisition (DIA) (39) modes.

## TIMS

**Fig. 2:**
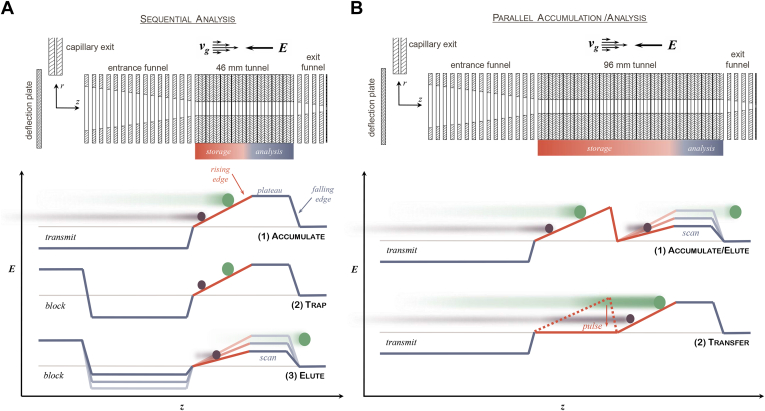
**Trapped ion mobility spectrometry (TIMS).***A*, operation principle of the single TIMS device. The *upper panel* shows the configuration of ion optical elements. Ions entering the vacuum through the glass capillary are deflected by 90° into the entrance funnel and focused into the TIMS tunnel. Mobility-separated ions are then sequentially released and refocused in the exit funnel for downstream mass analysis. *Arrows* indicate the direction of the forces from the gas flow (*v*_*g*_) and the electric field (*E*) working on the ions. The *lower panel* shows the electric field strength in the different steps of the TIMS analysis (accumulate, trap, and elute) as a function of the axial position (*z*). *B*, operation principle of the dual TIMS device. Same as *A*, with the difference that ion accumulation and mobility analysis are spatially separated in two parts of the longer TIMS tunnel. This allows parallel operation of both parts in time and increases the TIMS duty cycle close to 100% with a short transfer time from the accumulation to the analysis part. Reprinted from Silveira *et al.* (36) with permission from Elsevier.

From a standpoint of fundamental physics, the exact same principles apply to ions drifting through a stationary gas (as in drift tube IMS) as to the reverse, where stationary (*i.e.*, trapped) ions are immersed in a gas flow (40, 41, 42). To realize this concept, TIMS traps ions in an electrodynamic tunnel through which a gas flow is directed from the entrance to the exit at a pressure of about 2 to 3 mbar (Fig. 2*A*). The drag force on the ions because of the gas flow is roughly constant along the length of the device, but the drag is dependent on the ions' cross section—higher cross section yields a higher drag. This drag force is opposed by an analytical direct current (DC) field, which increases in strength along the tunnel. Ions entering the TIMS tunnel come to rest at an axial position at which the drag force on the ions (directed toward the tunnel exit) is counterbalanced by the force from the analytical field (directed toward the tunnel entrance). Because low-mobility ions require higher field strengths to counterbalance the drag from the gas, they are trapped further up the electric field gradient and thus further into the TIMS tunnel. Conversely, high-mobility ions come to rest closer to the entrance. After the desired accumulation time has been reached, further ions are prevented from entering the tunnel, and mobility analysis begins. During the analysis, ions are eluted according to their mobility by progressively lowering the strength of the analytical DC field. This is achieved by keeping the exit potential fixed and ramping the tunnel entrance potential at a constant and user-defined rate. However, because the entrance potential can be freely controlled, one can in principle program arbitrary scanning functions (43, 44).

Simulations of ion dynamics and numerical modeling have shown that most of the analytical separation is achieved at the electric field plateau as ions are released from the TIMS device (40, 41, 42). In analogy to drift tube IMS, in which ion mobility resolution increases with the drift length, the resolving power of TIMS scales with the effective drift length, which in this case is the length of the gas column passing by the ions (40). For this reason, a high ion mobility resolution (>100 *K/ΔK*) can be achieved even with a short physical length of the TIMS device and thus a comparably low potential difference, simply by reducing the ion mobility scan range or the rate of the release step (45, 46, 47, 48). For further details on the fundamentals of TIMS, please see also Ref. (35).

Assuming equal accumulation and ramp times, a single TIMS device cannot utilize more than half of the ions generated by a continuous ion source. To overcome this limitation, Silveira *et al.* (36) introduced an upstream ion trap by elongating the ion tunnel and dividing it into a trap, a transfer region, and a TIMS analyzer (Fig. 2*B*). In this “dual TIMS” configuration, the functions of accumulating ions and analyzing ions are separated in space. As a result, ions can be accumulated and analyzed in parallel in time. Ions are accumulated and trapped in the trap section but are then transferred in a single step to the TIMS analyzer, where they are subsequently mobility analyzed. In parallel, the trapping section is filled with the next batch of ions such that no ions have to be discarded given a sufficient trapping capacity. In practice, we have found an acquisition time of about 100 ms to be a good balance between filling the TIMS device to capacity and achieving the desired ion mobility resolution.

A more recent development is tandem TIMS (49). In this configuration, two TIMS analyzers are separated by an ion gate (44, 50) to transfer selected ion mobility ranges to the second TIMS. This opens up the possibility to perform collisional activation experiments of mobility-selected species at the interface, for example, to study protein unfolding or to further analyze fragment ions in the downstream TIMS. Tandem TIMS is thus of special interest for top–down proteomics, in particular if native-like protein structures are conserved (51, 52) but may also be applied in a similar manner to small molecules and peptides.

In connection with native, noncovalent, and top–down proteomics, ion heating, intentional or unintentional, is a topic of active research. In the study of protein unfolding, ion heating can be intentional, for example, in the case of collision-induced unfolding (51, 53). Collision-induced unfolding as a technique is still relatively new; however, early examples show it may provide insight, for example, in the study of protein–ligand binding (54). Unintentional ion heating can occur while injecting ions into, or while holding ions in, the TIMS analyzer (55, 56, 57). During injection, the DC field in the entrance funnel is of principle concern. Operating the entrance funnel at a low DC field strength reduces or eliminates signs of ion heating. In the TIMS tunnel, operating at a high rf amplitude or overfilling the TIMS analyzer with too many ions can lead to heating effects (47, 58). However, when operated properly—keeping potentials low and not overfilling—TIMS can be a powerful tool for a broad range of native applications (59, 60). Further opportunities may arise from the combination of ion fragmentation in or before TIMS (61, 62), followed by an MS^3^ analysis of the mobility-separated fragment ions with PASEF.

## The PASEF Principle

In MS/MS experiments with a TIMS–QTOF mass spectrometer, all precursor ions are accumulated simultaneously in the TIMS device and subsequently released in narrow ion mobility peaks of about 2 ms (full width at half maximum) within TIMS scans or “frames” that typically last 100 ms. This has the immediate advantage that the signal-to-noise ratio is increased because the signal is amplified by more than one order of magnitude as compared with continuous acquisition without TIMS (36). (Note that this poses additional challenges on the detector system, which the concentrated ion packages may saturate.) In addition, interfering background ions, including coeluting peptides of similar mass, are separated by ion mobility, which increases the relative fraction of fragment ions derived from the targeted precursor in the MS/MS spectrum. In line with this, Ogata and Ishihama (63) reported that, while currently limited to 1 Da-spaced reporter ions because of the instrument's mass resolution, TIMS can alleviate the ratio-compression problem of tandem mass tag–based quantification, at least to the same degree as MS^3^-based methods.

**Fig. 3:**
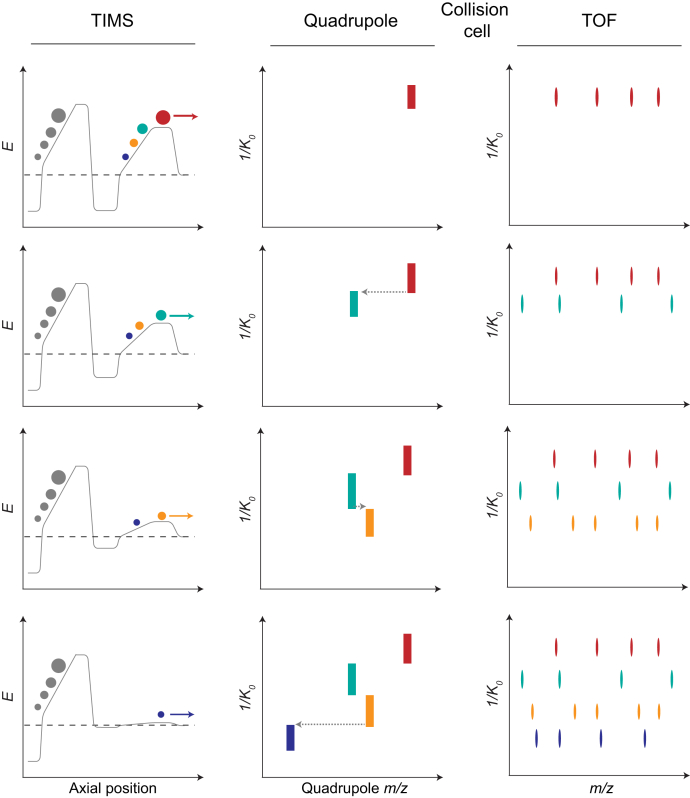
**Parallel accumulation–serial fragmentation (PASEF).** Time lapse (*top* to *bottom*) of a single PASEF scan on a TIMS quadrupole TOF mass spectrometer. All incoming ions are trapped simultaneously in the dual TIMS device (*left column*). As soon as the precursors of interest (color coded) are released from the TIMS device in the order of increasing ion mobility (decreasing *1/K*_*0*_), the position of the quadrupole isolation window switches rapidly to isolate multiple precursors sequentially for fragmentation of the precursor in the collision cell (*center column*). The fragment ions are mass analyzed with high-resolution TOF scans at the ion mobility position of their corresponding precursor ions (*right column*). *1/K*_*0*_, inverse reduced ion mobility; *E*, electric field strength; TIMS, trapped ion mobility spectrometry.

By their nature, conventional MS/MS experiments are highly inefficient. This is because only a small proportion of the total ion beam is selected for analysis, and the vast majority is discarded, especially in complex samples. However, this does not apply to PASEF because precursor ions in TIMS–QTOF instruments do not pass the analytical quadrupole in parallel (as in conventional MS/MS) but serially. As the signal of an individual precursor is compressed, its fragment ions are exclusively detected at the same TIMS elution time, and thus ion mobility position, of the precursor. This is the basis of the PASEF method, which positions the quadrupole isolation window as a function of the TIMS elution time (or that of any other time-dispersive IMS) (34). Rapidly switching the quadrupole position (<1 ms) allows selecting many precursors in a single TIMS scan (Fig. 3), thereby multiplying the efficiency of the MS/MS experiment by the number of selected precursors. In data-dependent mode, this factor depends on the ion mobility resolution as well as the quadrupole switching time and readily exceeds ten in complex samples (see also later) (29, 64). This 10× advantage comes without giving up the selectivity of mass selection and inherently decouples the ion accumulation step, and thus sensitivity, from the MS/MS acquisition rate. This is in contrast to conventional MS/MS methods, in which sensitivity decreases proportionally with the acquisition rate (65, 66).

## PASEF Scan Modes

**Fig. 4:**
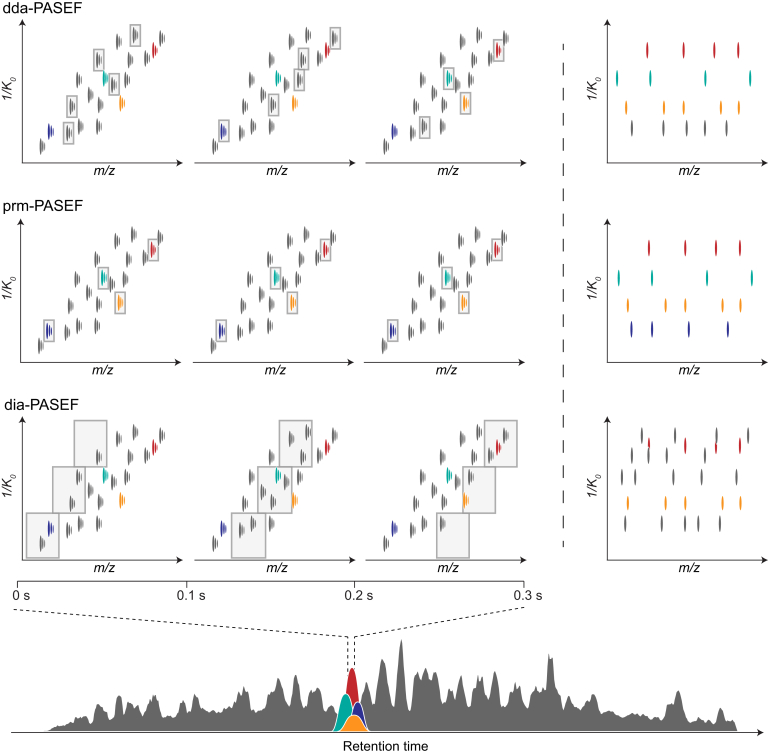
**PASEF scan modes.** Quadrupole isolation windows (*gray boxes*, *left panels*) in the two-dimensional *1/K*_*0*_—*m/z* plane for dda-, prm-, and dia-PASEF acquisition schemes with a 100 ms TIMS scan time. The PASEF MS/MS spectra (*right panels*) correspond to the precursor selection in the *third column*. dda, data-dependent acquisition; dia, data-independent acquisition; PASEF, parallel accumulation–serial fragmentation; prm, parallel reaction monitoring; TIMS, trapped ion mobility spectrometry.

The PASEF principle has been implemented in each of the three main acquisition strategies in the proteomics toolbox (Fig. 4). The most prevalent method to date, data-dependent acquisition (dda), aims to fragment as many selected precursors as possible to comprehensively characterize a biological sample of interest. As discussed previously, PASEF greatly increases the number of precursors that can be targeted in one experiment. We demonstrated that in a typical 2 h LC–MS experiment of a whole-cell proteome digest of a human cancer cell line over 600,000 MS/MS spectra can be acquired, which equates to a sequencing rate of >100 Hz throughout the entire elution time of peptides (29). Rather than allocating the entire sequencing capacity equally to distinct precursors, it is beneficial to sequence low-abundance precursors repeatedly (on average about two times) and aggregate the spectra in the postprocessing to increase signal-to-noise ratios. Across four replicate injections, and depending on the analysis software (see later), these were assigned to about 44,000 to 59,000 tryptic peptides, and up to ∼93,500 peptides when in addition considering semitryptic sequences (57). In contrast to a purely intensity-based *topN* approach, the online PASEF precursor scheduling algorithm in addition considers the two-dimensional separation of precursors to approximate optimal PASEF routes (*i.e.*, with the maximal number of precursors per cycle) in the *m/z versus* ion mobility plane. This maximizes the benefit of the PASEF principle and allows, for example, to exclude singly charged species by their relative ion mobility. Steigenberger *et al.* (67) extended this logic to include precursors of interest based on their CCS in a method termed caps-PASEF. The authors made use of the shifted distribution of monolinked and crosslinked peptides in the mass *versus* CCS space to bias their acquisition toward the more informative cross-linked peptides. As computational power increases, even more sophisticated algorithms and real-time search engines are conceivable to direct PASEF precursor selection efficiently within the available analysis time. We and others have already worked on similar concepts for Orbitrap mass spectrometers (68, 69, 70).

Rather than selecting precursors on the fly and only once, in targeted proteomics, the mass spectrometer is programmed to trace a predefined list of peptides over the expected elution time window (71, 72). A main limitation of targeted methods is that only a limited number of peptides can be monitored simultaneously, but this is greatly alleviated by the sequencing power of PASEF. For instance, implementing the PASEF principle, Lesur *et al.* (38) accurately quantified about 200 peptides spiked into a complex background proteome *via* parallel reaction monitoring-PASEF. Although the targeted precursors overlapped considerably in the 22-min chromatographic elution window, their separation by TIMS allowed to target on average four precursors per scan with no increase in cycle time. These results suggest that even faster chromatographic methods should be feasible, leading to an increased sample throughput with similar quantitative accuracy.

Molecular weight and ion mobility are correlated such that peptide ions with higher *m/z* generally have lower mobilities, that is, higher *1/K*_*0*_. In TIMS, they are released first, followed by ions of increasing mobility and typically decreasing *m/z*. We reasoned that following this characteristic profile with the analytical quadrupole could transmit the entire peptide ion cloud in a DIA mode (dia-PASEF) (39). In a first implementation, we stepped the quadrupole as a function of the TIMS elution time, which allowed to acquire close to 100% of the peptide fragment ion current in low-complexity samples. In full proteome digests, we increased selectivity by narrowing the isolation window and allocating the full precursor area to multiple and subsequent dia-PASEF scans. This provides an effective handle to tune the acquisition method, for example, to achieve shorter cycle times suited for fast chromatography or to analyze low sample amounts with high sensitivity. In a dramatic demonstration of this, combining the dia-PASEF principle with very low-flow chromatography (25–100 nL/min) and an experimental ion source with increased transmission, we were recently able to measure the proteomes of single cells sorted by fluorescence-activated cell sorting, with up to 1400 quantified proteins per cell (73). Even at this sensitivity, quantification was excellent (median coefficient of variation <10% for replicate injections of 1 ng digest), which we attribute partly to the reduction of interferences between peptides and the removal of noise *via* TIMS. We also note that advanced DIA schemes, such as scanning quadrupole (74, 75, 76) or overlapping-windowed (77) methods, should be readily compatible with dia-PASEF and could further improve the precursor selectivity while taking full advantage of the PASEF principle.

## Data Analysis

The additional ion mobility dimension and the extremely high number of raw data points present a challenge for data analysis. MaxQuant was amongst the first widely used proteomics tools to support dda-PASEF data (78, 79). It assembles four-dimensional MS^1^ features (*m/z*, retention time, ion mobility, and intensity) and automatically assigns the respective PASEF spectra to them based on precursor mass and mobility. Established mass recalibration routines have been extended to the extra dimension, allowing database searches with a ≤10 ppm precursor mass tolerance and resulting in median absolute peptide mass deviations of about 1 to 1.5 ppm. As an aside, these values are similar to the *impact II* QTOF instrument without TIMS (37), while—depending on transient lengths—Orbitrap instruments can achieve an even higher mass accuracy of about 0.5 ppm in proteomics. Although ion mobility is currently not taken into account for scoring peptide spectrum matches, aligning ion mobility values across experiments increases the confidence in “matching between runs” (transfer of identifications to LC runs where no fragmentation events are available) for label-free quantification. Through fragment ion and peak indexing, MSFragger and the accompanying IonQuant achieve several-fold faster processing times, which renders semispecific and so-called open database searches practical to perform (57, 80). The same group also implemented a variant of the matching between runs algorithm that feeds, amongst others, ion mobility data into a machine learning model to discriminate true and false matches (81). In addition to academic tools, commercial software also supports TIMS data increasingly, for example, PEAKS (82) or SpectroMine. Our own group has developed a tool called AlphaTIMS (83), which introduces an extremely efficient data structure in the Python language that can be used as the basis of visualization and processing the full detail of the raw data, without requiring aggregating it up front (see also openTIMS for an effort in the same direction (84)). AlphaTIMS is part of the open source AlphaPept framework that allows robust and fast processing also of timsTOF data (85).

Targeted proteomics data acquired with parallel reaction monitoring-PASEF can be processed with Skyline (86), which also supports proteome-wide analysis of dia-PASEF experiments. The original dia-PASEF publication detailed the extension of the targeted data analysis principle for DIA (87) to the new data format and the development of the open source “Mobi-DIK” toolkit as part of the OpenSWATH (88) environment (39). In the data processing, the ion mobility dimension is used to restrict the data extraction window as well as to extract several mobility-based scores, which increased the number of identified precursors by >20% as compared with the naive analysis of an exemplary HeLa dataset. Spectronaut (4) offers a popular commercial alternative for library-based and library-free analysis. The more recently developed DIA-NN software (89) uses neural networks to match the MS/MS data to a library and has recently been extended for dia-PASEF. Reanalysis of our original dia-PASEF data with DIA-NN and a library generated by MSFragger significantly increased protein identifications while improving quantification, including the identification of more than 5000 proteins in 5-min EvoSep runs (90). With MaxDIA, dia-PASEF data can now also be analyzed within the MaxQuant environment (91). All in all, there is a growing number of software solutions available for TIMS–PASEF data, which can only foster a more widespread application of the technology and accelerate its development.

## The “Perfect Data Cuboid” Generated by TIMS

**Fig. 5:**
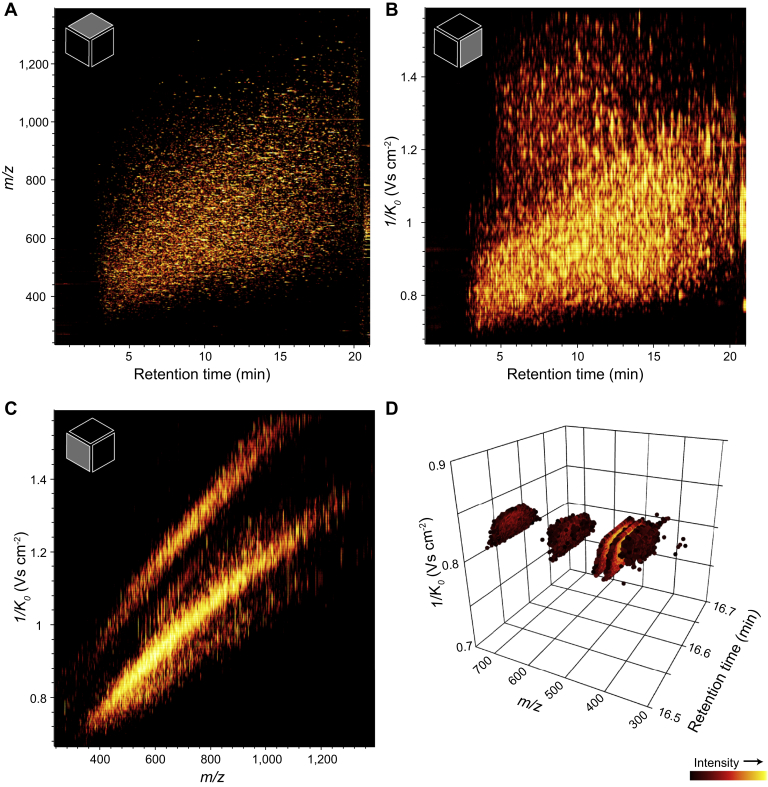
**The TIMS data cuboid.***A*–*C*, AlphaTims (83) visualization of the about 2.4 million most abundant raw data points on the MS^1^ level from a 21-min dda-PASEF analysis of a HeLa digest. *A*, projection on the retention time—*m/z* plane. *B*, projection on the retention time—ion mobility plane. *C*, projection on the *m/z*—ion mobility plane. *D*, fragment ions of a single peptide precursor from a tryptic bovine serum albumin digest acquired with a high duty cycle dia-PASEF method. See supplemental data for an interactive version and the corresponding precursor ion trace. dda, data-dependent acquisition; dia, data-independent acquisition; PASEF, parallel accumulation–serial fragmentation; TIMS, trapped ion mobility spectrometry.

It is interesting and insightful to characterize the multidimensional data space in more detail. Despite the general trend of correlation of mass and mobility, recent studies demonstrated that TIMS extends the peptide separation space by at least one quarter of the theoretical maximum for a fully orthogonal separation, which translates into an over 10-fold increase in analytical peak capacity (63, 92). Retention time, ion mobility, and *m/z* span a data cuboid, in which each peptide is positioned with a distinct uncertainty in each dimension (Fig. 5, *A*–*C*). In typical proteomics experiments, the calibrated position in *m/z* can be defined with a median absolute accuracy of <1.5 ppm (29, 79). In contrast, peptide retention times can shift minutes between experiments, and even on the same column and instrument, nonlinear alignment can be required to localize a peptide within better than 1 min of a 120-min gradient. Because TIMS is a gas phase separation technique primarily controlled by electric potentials, a simple linear alignment can be sufficient to account for changes in the gas flow causing drifts in the measured ion mobility value over time. Illustrating this point, we observed a median coefficient of variation of 0.4% in a dataset of 168 LC–TIMS–MS experiments acquired on multiple instruments and over several months (92). In that study, we also estimated that determining the mobility position of a precursor ion within ∼1% accuracy reduces the number of peptide candidates in a ±1.5 ppm mass window by a factor of 2 to 3. Note that dia-PASEF not only positions all precursors in the data cuboid but also detects each fragment ion precisely at the ion mobility position of the corresponding precursor ions (Fig. 5*D*). Interestingly, with a duty cycle approaching 100%, the data density increases and becomes virtually complete. We therefore refer to this as an “ideal data cuboid” because it contains, in principle at least, a complete record of all MS/MS information that can be obtained from a given sample.

A more fundamental research interest in IMS is the structure of ions in the gas phase, which can be derived from measured ion mobility values by means of collisional, or more precisely momentum transfer, cross sections (CCS, Ω) (12, 93). Because TIMS shares the underlying physics with classical drift tube IMS as already mentioned, it should be feasible to derive ion mobility and hence CCS values from first principles (40, 42). In practice, however, a linear model is commonly used to calibrate TIMS elution voltages with known *1/K*_*0*_ values (40, 46, 94). In combination with PASEF, this allows to measure CCS values on a very large scale as we have recently demonstrated with a set of 2.5 million peptide spectrum matches (and hence CCS measurements) from over 400,000 unique peptide sequences (92). This revealed, on a global scale, sequence-specific determinants of CCS values, such as hydrophobicity and the position of basic residues. Affirming the contribution of the amino acid sequence, Chang *et al.* (95) modeled peptide ^TIMS^CCS values with sequence-specific “intrinsic size parameters”. As an alternative, we found the size of our dataset to be sufficient to train a deep learning model that predicts CCS values based solely on the linear amino acid sequence and charge state (92). A surprising finding was that the accuracy of the model reached a plateau beyond ∼200,000 training values, suggesting that a combination of more precise data and even more sophisticated machine learning models (96) may be necessary to improve predictions. Already, the current accuracy fits many practical purposes, such as the generation of *in silico* libraries for DIA. Another challenge arises from the well known but in larger datasets often neglected fact that peptides can adopt multiple conformations in the gas phase, each with distinct CCS values (97, 98).

## Outlook

Despite its relatively short history of development, TIMS–PASEF is becoming a widely used technology in proteomics laboratories. Their high-sequencing speeds should make PASEF scan modes particularly attractive for emerging high-throughput applications of proteomics that capitalize on fast LC separations (89, 99, 100). As an illustration of this point, the increased throughput and robustness allows drastic upscaling of protein–protein interaction screens (101). Similarly, we expect researchers to leverage the intrinsic sensitivity of the technology. Zaro *et al.* (102), for example, used dda-PASEF to study the proteomes of young and old mouse hematopoietic stem cells and their progenitors, including rare cell types of which only few thousands to ten thousands can be purified from a single mouse. Analyzing the proteomes of fluorescence-activated cell sorting–purified human β-cells, Fu *et al.* (103) revealed a link between glucose metabolism and cellular sensitivity to inflammation. Examples for the contribution of the PASEF technology to research during the global pandemic of the coronavirus disease 2019 include the proteomics analysis of patient-derived formalin-fixed paraffin-embedded lung tissue (104) as well as blood serum (105) and urine (106) samples.

As TIMS and PASEF are entering routine use, ongoing developments in both hardware and software are further expanding its capabilities. Recently, Jeanne Dit Fouque *et al.* (107) introduced a new TIMS design with convex electrodes that generates a higher pseudopotential and thereby extends the analytically accessible mass and mobility range up to macromolecular assemblies such as RNA polymerase or the 801 kDa GroEL complex. As mentioned previously, using a brighter ion source and a TIMS design with increased charge capacity, we developed an ultrahigh sensitivity workflow for label-free quantitative proteomics of single human cancer cells (73). This study clearly demonstrated the benefit of ion mobility in separating background interferences from peptide signals, which should also transfer to other sample-limited scenarios such as the analysis of microdissected tissue samples (108) or post-translational modifications (109).

TIMS and PASEF provide efficient access to large-scale CCS measurements, allowing to characterize the multidimensional data space in unprecedented detail (92). As CCS values are determined by the amino acid sequence, it will be interesting to extend these studies to further peptide classes, for example, cross-linked or modified peptides, in particular given the prospect of separating positional isomers in the gas phase (67, 110, 111, 112). We believe that emerging deep learning techniques will propel this development to make full use of the additional information (113).

We conclude that TIMS–PASEF holds great promise for proteomics research. In some ways, this technology is just at its beginnings, and as the community grows and the technology continues to advance, more of its untapped potential will be unleashed. We also note that many of the advantages readily transfer to other biological compound classes, including metabolites and lipids (64, 114, 115).

## Supplemental data

This article contains supplemental data.

## Conflict of interest

M. A. P. is an employee of Bruker, who manufactures the timsTOF mass spectrometer. All the other authors declare no competing interests.

## Supplemental Data

BSA_3d_ms1.html

BSA_3d_ms2.html
